# Susceptibility to caspofungin is regulated by temperature and is dependent on calcineurin in *Candida albicans*

**DOI:** 10.1128/spectrum.01790-23

**Published:** 2023-11-15

**Authors:** Lijun Zheng, Yi Xu, Chen Wang, Feng Yang, Yubo Dong, Liangsheng Guo

**Affiliations:** 1 Department of Ultrasound Medicine, The Second Affiliated Hospital of Soochow University, Suzhou, China; 2 Department of Pharmacy, The 960 Hospital of PLA, Jinan, China; 3 Department of Pharmacology, Shanghai Tenth People’s Hospital, Tongji University School of Medicine, Shanghai, China; 4 Department of Obstetrics and Gynecology, The Second Affiliated Hospital of Soochow University, Suzhou, China; Universidade de Sao Paulo, Sao Paulo, Brazil

**Keywords:** *Candida albicans*, caspofungin, temperature, calcineurin

## Abstract

*Candida albican*s is a prevalent opportunistic human fungal pathogen. Echinocandins are first-line drugs for the treatment of invasive candidiasis. Molecular mechanisms of resistance to echinocandins have been extensively studied; however, the effect of physiological factors on antifungal efficacy of echinocandins is unexplored. Here, we found temperature-modulated susceptibility to caspofungin in medium-independent and strain background-independent manner in *C. albicans*. Deletions of protein kinase C (PKC) pathway and calcineurin-Crz1 signaling pathway genes conferred hypersensitivity to caspofungin, but only deletion or pharmacological inhibition of calcineurin subunits inverted the temperature effect on susceptibility to caspofungin. Furthermore, the enhanced growth at lower temperature was not due to altered expression of some established genes such as *FKS*, *CHS*, or *CHT* genes; PKC or calcineurin pathway genes; or *HSP90*. The expressions of other heat shock protein genes, including *HSP12* and *HSP70*, were higher at lower temperature. We posit that the temperature-modulated susceptibility to caspofungin is a non-canonical mechanism that is dependent on heat shock proteins and calcineurin.

**IMPORTANCE:**

Echinocandins are the newest antifungal drugs and are first-line treatment option for life-threatening systemic infections. Due to lack of consensus regarding what temperature should be used when evaluating susceptibility of yeasts to echinocandins, typically either 30°C, 35°C, or 37°C is used. However, the impact of temperature on antifungal efficacy of echinocandins is unexplored. In the current study, we demonstrated that *Candida albicans* laboratory strain SC5314 was more susceptible to caspofungin at 37°C than at 30°C. We also found that calcineurin was required for temperature-modulated caspofungin susceptibility. Surprisingly, the altered caspofungin susceptibility was not due to differential expression of some canonical genes such as *FKS*, *CHS*, or *CHT* genes. The molecular mechanism of temperature-modulated caspofungin susceptibility is undetermined and deserves further investigations.

## INTRODUCTION

In recent years, due to steady increase of immunocompromised populations, opportunistic fungal infections are emerging as an important public health concern and cost burden (1). *Candida* spp are the most common human fungal pathogens and are the fourth leading cause of nosocomial bloodstream infections in the United States (2). Among them, *Candida albicans* is the most common fungal pathogen responsible for nosocomial systemic infections and the most commonly isolated pathogen from clinical samples obtained from mucous membranes (3). *C. albicans* is a harmless commensal colonizer dwelling in human oral cavity, gastrointestinal tract, and vaginal. However, this fungus is also the leading pathogen causing infections that range from superficial infections of the skin to life-threatening systemic infections, especially in immunocompromised populations (3). In 2022, the World Health Organization released the first-ever list of health-threatening fungi. *C. albicans* is among the four members of the most dangerous “critical group” (4).

Currently, only four classes of antifungal drugs are available: azoles, polyenes, 5-flucytosine, and echinocandins. Echinocandins are the most optimal first-line antifungal agents for the treatment of invasive candidiasis (5). Three echinocandin drugs are approved by the United States Food and Drug Administration to treat candidemia: caspofungin (CSP), anidulafungin (ANF), and micafungin (MCF). Echinocandins are fungicidal against *Candida* spp and fungistatic against *Aspergillus* spp but are inactive against *Cryptococcus* spp (6). Echinocandins act by non-competitively binding to β-(1,3)-glucan synthase (7). Inhibition of the major fungal cell wall component β-(1,3)-glucan biosynthesis leads to growth inhibition or death owing to imbalance in osmotic pressure (8).

The incidence of CSP resistance is rising in recent years (9–11). Resistance to CSP is usually due to point mutations in *FKS* genes, which encode subunits of the β-(1,3)-glucan synthase (12). Mutation usually occurs in two highly conserved hotspot regions of *FKS* genes encompassing amino acids 641–649 and amino acids 1357–1364 (13). The amino acids of *FKS* mutation decrease the sensitivity of glucan synthase to echinocandins and cause cross-resistance to various echinocandins (9, 14, 15). About 80% mutation in *FKS1/GSC1* of *C. albicans* occur in Ser645 and Phe641, which brings the most prominent resistance phenotype (16, 17). The roles of *FKS2/GSL1* and *FKS3/GSL2* in resistance to CSP in *C. albicans* are largely unknown; however, deletions of *FKS2* and *FKS3* can upregulate expression of *FKS1*, increase cell wall glucan content, and decrease susceptibility to echinocandins (18). In *glabrata*, mutations occur more frequently in *FKS2* (19). In *auris*, S639F mutation of *FKS1* is associated with CSP resistance in clinical isolates (20).

In addition to *FKS* mutations, cell wall salvage mechanism also contributes to CSP resistance. Fungal cell wall consists of glucan, chitin, and glycoproteins. Inhibition of glucan biosynthesis usually results in compensatory increase of chitin synthesis (21, 22). Both *in vitro* and *in vivo*, elevated chitin confers CSP resistance in *C. albicans* (23, 24). Moreover, *C. albicans* clinical isolates containing *FKS1* mutation generally have higher chitin levels (23). Decreased digestion of chitin, due to loss-of-function mutation in chitinase genes *CHT2* and *CHT3* (25), and decreased copy number of *CHT2* also confer CSP resistance in *C. albicans* (26).

Compensatory increase of chitin in response to CSP treatment is dependent on the regulatory circuit composed of PKC pathway, calcineurin pathway, high-osmolarity glycerol pathway, and heat shock protein 90 (Hsp90) (22, 27). Deletion of PKC pathway genes, including *PKC1*, *BCK1*, *MKK2,* and *MKC1*, or genes encoding downstream transcription factors, including *SWI4*, *SWI6* and *RLM1*, causes hypersensitivity to echinocandins (28, 29). Pharmacological inhibition of calcineurin is synergistic with echinocandins against clinical isolates harboring *FKS1* mutations (30). Genetic deletion of *CMP1* and *CNB1*, which encodes the catalytic subunit and regulatory subunit of calcineurin, respectively, or *CRZ1*, which encodes the major downstream transcription factor of the calcineurin pathway, can enhance sensitivity of *C. albicans* to echinocandins (29, 30). Calcineurin is the client protein of Hsp90, a highly conserved molecular chaperone. Hsp90 plays a pivotal role in activation and stabilization of calcineurin (30).

Although genes and regulatory networks required for CSP resistance have been studied extensively, physiological factors affecting antifungal efficacy of CSP are largely unexplored. Recent studies indicate that temperature affects antifungal potency of azoles against *C. albicans* (31, 32); however, the impact of different temperatures on the antifungal effect of CSP is still unknown. There is no consensus among researchers regarding what temperature should be used in yeast studies. Usually either 30°C or 37°C is used. In the current study, we found that *C. albicans* was less susceptible to CSP at 30°C than at 37°C. The temperature effect was independent on medium composition, or genetic backgrounds of test strains, and was independent on the PKC pathway, albeit deletion of PKC pathway genes that caused hypersensitivity to CSP. Pharmacological inhibition or genetic knockdown of calcineurin subunits inverted temperature effect on CSP susceptibility; however, the transcription factor of the calcineurin pathway, Crz1, was not involved. Comparison of transcriptomes of cells grown at 30°C and 37°C indicated that several genes encoding chaperone proteins were more abundant at 30°C. We posit that heat shock proteins and the client protein calcineurin are required for temperature-modulated CSP susceptibility in *C. albicans*.

## MATERIALS AND METHODS

### Strains and growth conditions

The strains used in this study are listed in Table S1. The stock culture was preserved in 25% of glycerol and maintained at −80°C. Cells were routinely grown in the yeast extract–peptone–dextrose (YPD) media [1% (wt/vol) yeast extract, 2% (wt/vol) peptone, and 2% (wt/vol) D-glucose] at 37°C in a shaking incubator at 150–200 rpm. For solid medium, 2% [wt/vol] agar was added. SD agar plates [0.67% (wt/vol) yeast nitrogen base without amino acids, 2% (wt/vol) D-glucose, and 2% (wt/vol) agar] and SDC agar plates [0.67% (wt/vol) yeast nitrogen base without amino acids, 0.2% of all amino acids mixture, 2% (wt/vol) D-glucose, and 2% (wt/vol) agar] were used. The same medium was used for growing cells and doing tests. The medium and temperature used in each experiment are specified in the figure legends. Drugs were dissolved in dimethyl sulfoxide (DMSO) and stored at −20°C. For the selection of gene knockout strains, YPD agar containing 400 µg/mL nourseothricin (NAT; Werner BioAgents) medium was used (YPD + NAT). To evict the disruption cassette, we used yeast nitrogen base (YNB)–bovine serum albumin (BSA) [0.17% (wt/vol) YNB, 2% (wt/vol) D-glucose, 0.02% (wt/vol) BSA, and 2% (wt/vol) agar] plates.

### Spot assay

Cells were suspended in distilled water and adjusted to 1 × 10^7^ cells/mL. A total of 3 µL of 10-fold serial dilutions were spotted on YPD, SD, or SDC plates with or without drugs (control) at 30°C or 37°C and photographed after 2 days. Medium and temperature were indicated in the figures.

### Deletions

Gene deletions were performed as described previously (31). Primers are the same as described previously (31). Plasmid pJK863 (33) was used as template for amplifying the NAT1 flipper gene deletion cassette. Approximately 500 bp regions flanking the CDS of the target gene to be deleted were amplified using the genomic DNA of YJB-T490 as the template. The upstream region of the gene was fused by PCR to the 5′ region of the cassette, and the downstream region of the gene was fused to the 3′ region of the cassette. The upstream and downstream fusion products for each gene were then simultaneously transformed in *C. albicans* by following the lithium acetate method (34). Transformants were selected on YPD plates supplemented with 400 µg/mL NAT. Diagnostic PCR using primers that annealed outside the flanking homologous regions of the gene was performed to confirm the replacement of the gene with the *NAT1* flipper cassette. The *NAT1* flipper was evicted by streaking the clones on YNB–BSA plates.

### Growth curve

Approximately 1 × 10^3^ cells/mL of each strain were suspended in YPD broth and 150 µL was transferred to a 96-well plate. Optical density at 595 nm (OD_595_) was measured every 15 min for 24 h at 30°C using a Tecan plate reader (Infinite F200 PRO; Tecan, Switzerland).

### RNA-Seq

RNA-seq was performed as described previously (29). SC5314 was inoculated to a starting OD_600_ of 0.2 in 50 mL of YPD broth. The culture was incubated in a shaker at 30°C and 37°C until the OD_600_ reached 1.0. Cultures were collected by centrifugation (comparison between 30°C and 37°C) or divided into two batches: one batch was supplemented with 100 ng/mL CSP, and the other batch was supplemented with an equal volume of DMSO. Three hours later, the cultures were collected by centrifugation, washed, and flash frozen in liquid nitrogen. The total RNA was extracted for six independent samples, corresponding to two conditions and three biological replicates. Total RNA extraction and purification, library construction, and sequencing were performed as described in Yang et al. (35). Raw sequence files (.fastq files) underwent quality control analysis using the FastQC tool (http://www.bioinformatics.babraham.ac.uk/projects/fastqc). Reads were mapped to the *C. albicans* SC5314 reference genome (http://www.candidagenome.org/download/sequence/C_albicans_SC5314/Assembly22/current/). Differential gene expression profiling was carried out using DESeq2 (36) with standard parameters. Genes with false discovery rate-adjusted *P*-value (<0.05) and expression fold changes of more than 1.5 or less than −1.5 were considered differentially expressed.

## RESULTS

### Temperature modulates echinocandins susceptibility

**Fig 1:**
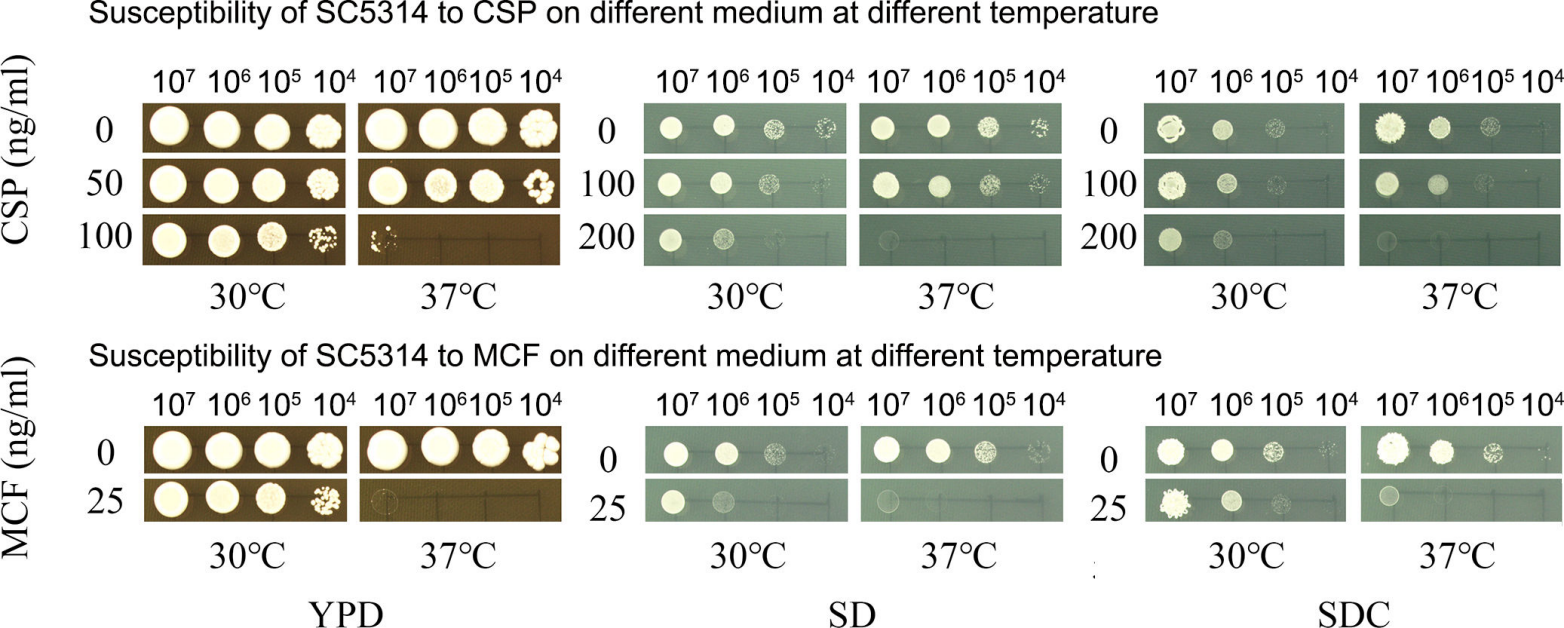
Temperature modulates resistance to echinocandins. Spot assays were performed using YPD, SD, and SDC media. Cells were suspended in distilled water and adjusted to 1 × 10^7^ cells/mL; 3 µL of 10-fold serial dilutions was spotted on the plates. The plates were supplemented with caspofungin (CSP, top panel) or micafungin (MCF, bottom panel). Drug concentrations are indicated in the figure. The plates were incubated for 48 h at 30°C and 37°C, as indicated in the figure.

Susceptibility of the *C. albicans* reference strain SC5314 to CSP and MCF was measured at two different temperatures using different media. On YPD, the growth was not inhibited by 100 ng/mL CSP (Fig. 1, top panel) or 25 ng/mL MCF (Fig. 1, bottom panel) at 30°C, but the growth was completely inhibited at these drug concentrations at 37°C. On SD and SDC, the inhibition of growth by 200 ng/mL of CSP (Fig. 1, top panel) or 25 ng/mL MCF (Fig. 1, bottom panel) was more obvious at 37°C than at 30°C. Thus, independent on medium composition, SC5314 is generally less susceptible to CSP and MCF at 30°C than at 37°C.

### Temperature effect on CSP susceptibility is independent on PKC

**Fig 2:**
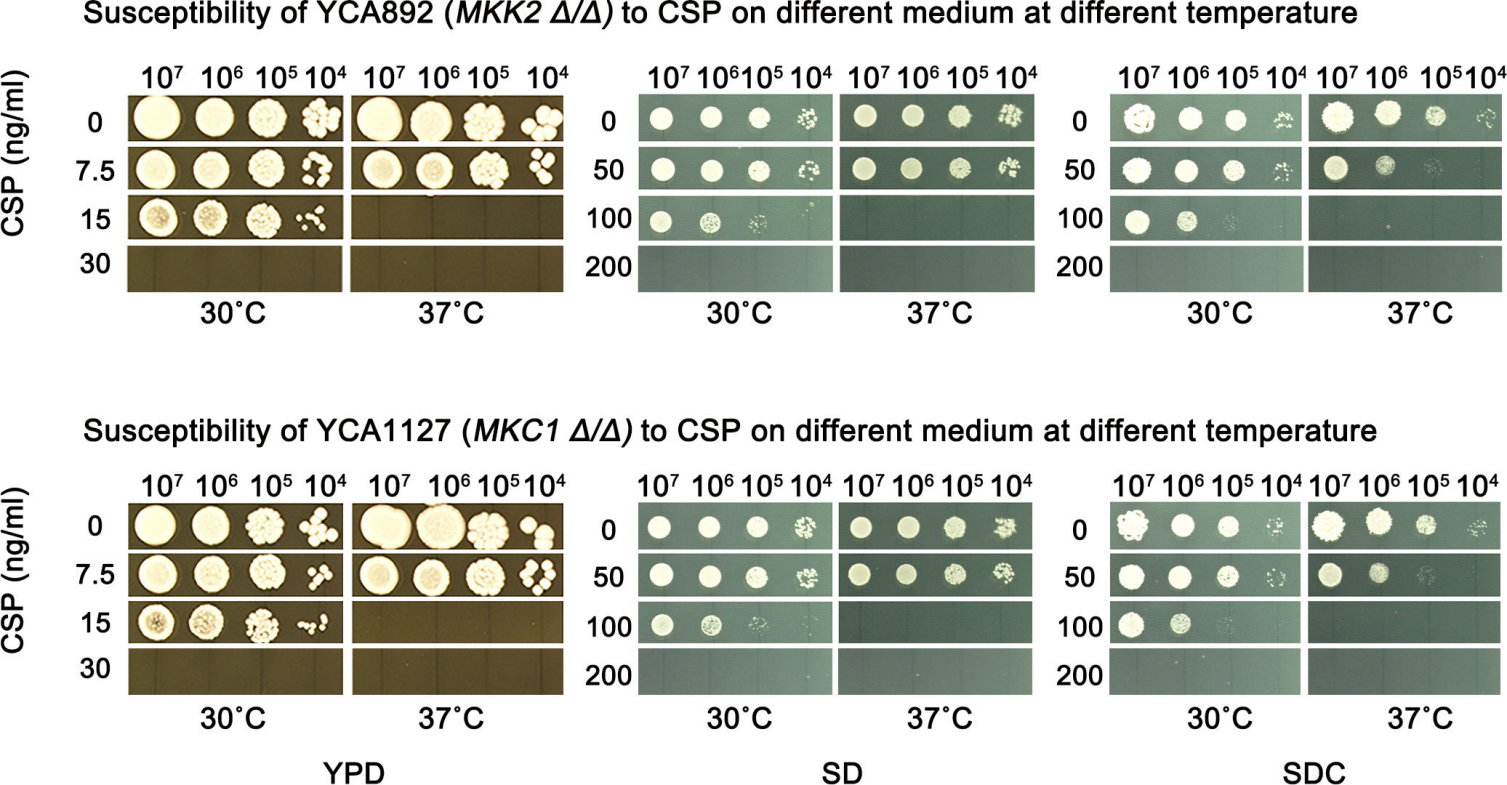
Role of PKC pathway in temperature-modulated caspofungin resistance. SC5314-derived strains with deletions of *MKK2* (top panel) and *MKC1* (bottom panel) were evaluated with spot assay. The media, temperatures, and drug concentrations are indicated in the figure. For each strain, the cell densities spotted on the plates were 1 × 10^7^, 1 × 10^6^, 1 × 10^5^, and 1 × 10^4^ cells/mL. The plates were incubated for 48 h then photographed.

We asked if intact PKC pathway was required for temperature-modulated CSP susceptibility. Knockouts of PKC pathway genes *MKK2* and *MKC1* were evaluated at 30°C and 37°C using three different media. As shown previously in Fig. 1, at 30°C, on YPD, SD, and SDC, wild-type strain could grow in the presence of 100 ng/mL, 200 ng/mL, and 200 ng/mL of CSP, respectively (Fig. 1, top panel), while growths of *mkk2 Δ/Δ* strain (Fig. 2, top panel) and *mkc1 Δ/Δ* strain (Fig. 2, bottom panel) were completely inhibited by 30 ng/mL, 200 ng/mL, and 200 ng/mL of CSP, respectively. At 37°C, on YPD, SD, and SDC, wild-type strain could grow in the presence of 50 ng/mL, 100 ng/mL, and 100 ng/mL of CSP, respectively (Fig. 1, top panel), while growths of *mkk2 Δ/Δ* strain (Fig. 2, top panel) and *mkc1 Δ/Δ* strain (Fig. 2, bottom panel) were completely inhibited by 15 ng/mL, 100 ng/mL, and 100 ng/mL of CSP, respectively.

Comparing between 30°C and 37°C, on YPD, SD and SDC, both *mkk2 Δ/Δ* and *mkc1 Δ/Δ* strains were less susceptible to CSP at 30°C than at 37°C (Fig. 2). Therefore, deletion of *MKK2* or *MKC1* caused hypersensitivity to CSP at both 30°C and 37°C; however, in deletion strains, lower temperature (30°C) still enables better adaptation to CSP than higher temperature (37°C).

### Temperature effect on susceptibility to CSP is dependent on calcineurin

The calcineurin-Crz1 signaling pathway is conserved across multiple pathogenic fungi. Calcineurin is a major player in calcium^2+^-dependent signal transduction pathways of eukaryotes. The transcription factor Crz1 is the major effector of calcineurin (37). Calcineurin consists of a catalytic subunit and a regulatory unit, which is encoded by *CMP1* and *CNB1*, respectively, in *C. albicans* genome (37).

In this study, the role of calcineurin-Crz1 signaling pathway in temperature-modulated CSP susceptibility was evaluated in two genetic backgrounds: SC5314 and YJB-T490. Two methods were employed: genetic deletions of *CMP1*, *CNB1*, and *CRZ1* and pharmacological inhibition of calcineurin.

**Fig 3:**
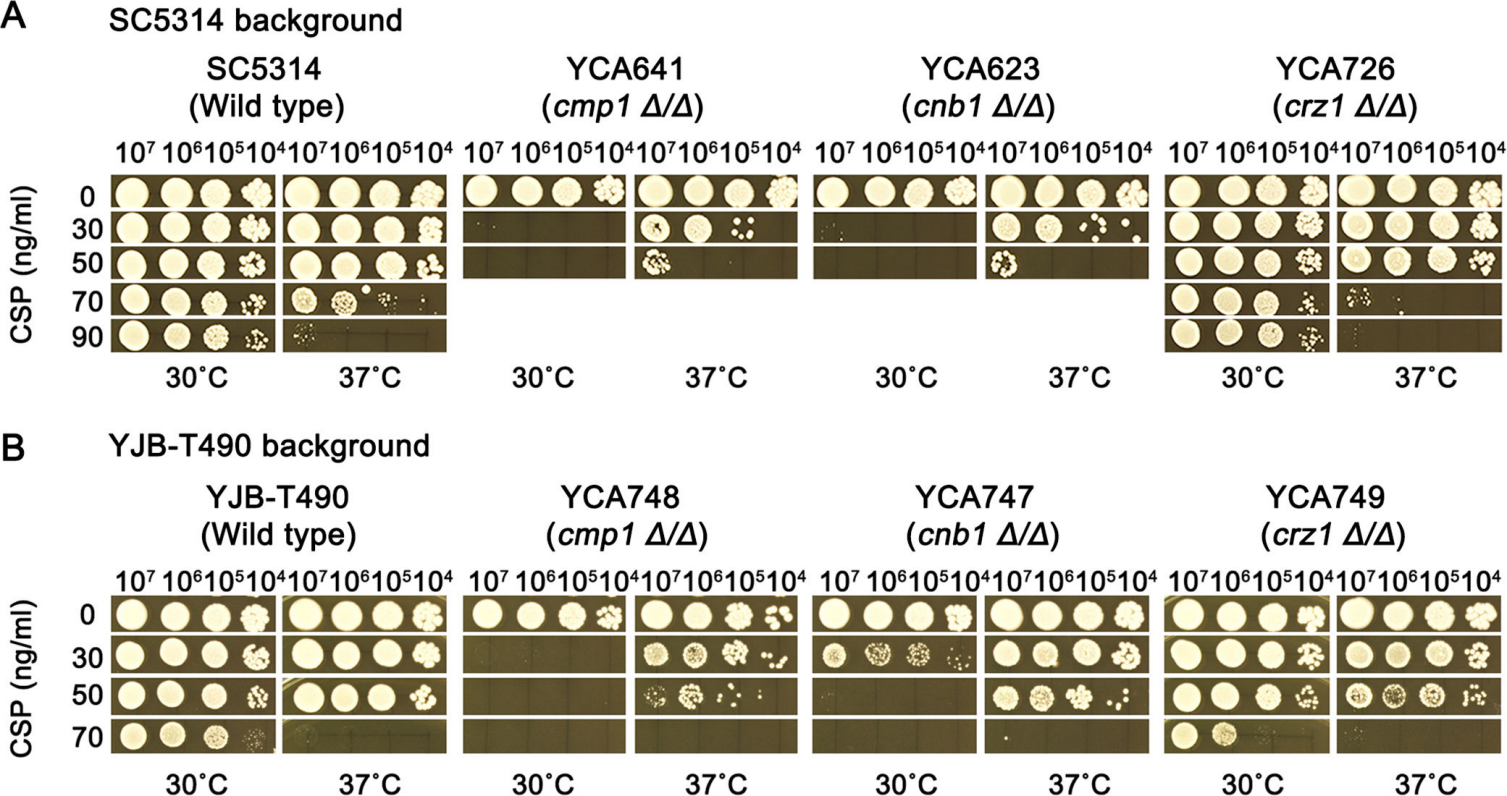
Role of calcineurin-Crz1 pathway in temperature-modulated caspofungin resistance. In SC5314 (top panel) and YJB-T490 (middle panel), *CMP1*, *CNB1,* and *CRZ1* were deleted. Wild-type and deletion strains were tested with spot assay on YPD-agar medium supplemented with caspofungin (CSP). The wild-type strains were also tested with spot assay using YPD-agar supplemented with calcineurin inhibitor cyclosporin A (CsA, 0.5 µg/mL) (bottom panel). The temperatures and drug concentrations are indicated in the figure. The plates were incubated for 48 h then photographed.

In SC5314 background, at 30°C, the wild type was not inhibited by 90 ng/mL CSP, while *cmp1Δ/Δ* and *cnb1Δ/Δ* strains were completely inhibited by 30 ng/mL CSP. However, *crz1Δ/Δ* strain was not inhibited by 90 ng/mL CSP. At 37°C, the wild type was not inhibited by 50 ng/mL CSP, while *cmp1Δ/Δ* and *cnb1Δ/Δ* strains were obviously inhibited by 50 ng/mL CSP, but the *crz1Δ/Δ* strain was also not inhibited by 50 ng/mL. Comparing between 30°C and 37°C, interestingly, both *cmp1Δ/Δ* and *cnb1Δ/Δ* strains were less susceptible at 37°C than at 30°C; however, similar to wild type, the *crz1Δ/Δ* strain was still more susceptible at 37°C than at 30°C (Fig. 3A).

In YJB-T490 background, at 30°C, the wild type was not inhibited by 70 ng/mL CSP, while the *cmp1Δ/Δ* and *cnb1Δ/Δ* strains were completely inhibited by 30 ng/mL and 50 ng/mL CSP, respectively. At 37°C, the wild type was not inhibited by 50 ng/mL CSP and was completely inhibited by 70 ng/mL CSP, while the *cmp1Δ/Δ* and *cnb1Δ/Δ* strains were all completely inhibited by 50 ng/mL CSP. However, *crz1Δ/Δ* strain had similar extent of CSP susceptibility to wild type at both 30°C and 37°C. Compared between 30°C and 37°C, both *cmp1Δ/Δ* and *cnb1Δ/Δ* strains were less susceptible to CSP at 37°C than at 30°C, while the *crz1Δ/Δ* strain was still more susceptible at 37°C than at 30°C (Fig. 3B). We also measured growth curves of deletion strains and the wild types in YPD broth without CSP. We found that all strains had similar growth curves (Fig. S1); thus, altered CSP susceptibility in the deletion strains was not due to change of growth caused by deletions.

Requirement of calcineurin for temperature-modulated CSP susceptibility was also evaluated by using calcineurin inhibitor cyclosporin A (CsA). In the YPD medium supplemented with 0.5 µg/mL CsA, both SC5314 and YJB-T490 were less susceptible to CSP at 37°C than at 30°C (Fig. 3C). As a control, CsA at 0.5 µg/mL was not inhibitory to the strains (Fig. S2).

Taken together, in both SC5314 and YJB-T490 backgrounds, calcineurin is required for temperature-modulated CSP susceptibility. Genetic deletion or pharmacological inhibition of calcineurin inverted extent of CSP susceptibility at 30°C and 37°C.

### Canonical genes associated with CSP susceptibility are not involved in temperature-modulated CSP susceptibility

We asked why susceptibility to CSP was lower at 30°C than at 37°C. Transcriptome analysis indicated that some genes were regulated by CSP similarly 30°C and 37°C. For example, the *FKS* gene *GSC1*; the *CHS* genes *CHS2*, *CHS3*, *CHS4*, and *CHS7*; and the calcineurin-Crz1 signal pathway genes *CMP1* and *CRZ1* were upregulated by CSP at both temperatures. The *CHT* gene *CHT3* and the *HSP* genes *HSP21*, *HSP70*, *HSP104*, and *HSP12* were downregulated by CSP at both temperatures. Some genes were differentially regulated by CSP at 30°C and 37°C. For example, the PKC pathway genes *BCK1* and *RLM1* were upregulated by CSP at 30°C but not at 37°C, while the *HSP* genes *HSP31* and *HSP104* were downregulated by CSP at 30°C but not at 37°C (Table S2).

**Fig 4:**
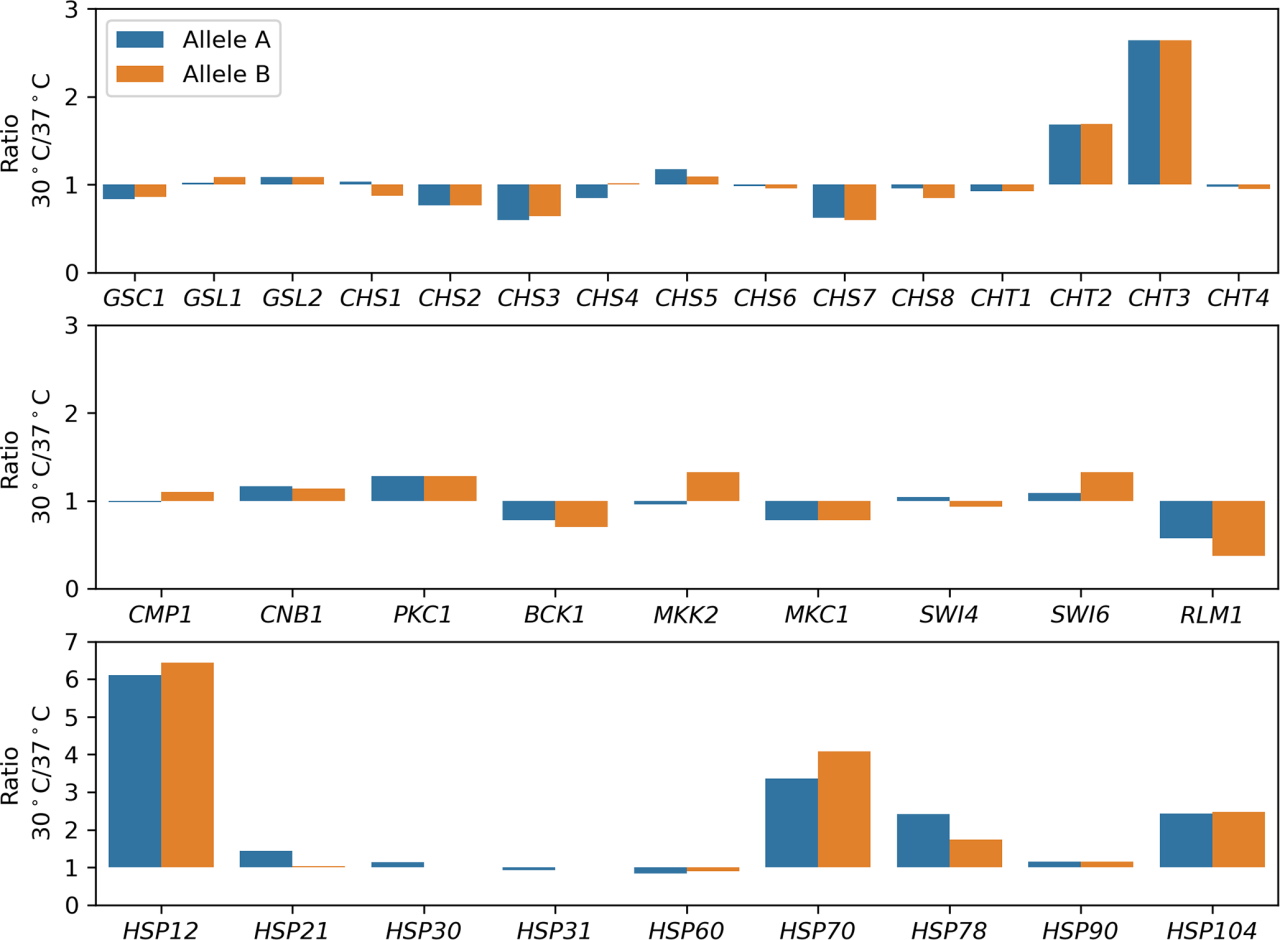
Transcriptomes of cells growing at different temperatures. SC5314 was grown in YPD broth to log phase at 30°C and 37°C. Ratios of gene expression levels were calculated by dividing the transcript abundance under 30°C by that under 37°C. For each gene, ratios of both alleles (allele A and allele B) were calculated.

We compared transcriptomes of log phase cells of SC5314 incubated at 30°C and at 37°C. Genes with ratios of transcripts at 30°C vs 37°C higher than 1.5 and lower than 0.67 were considered higher and lower expressed, respectively. We found that cells did not have altered expression of *FKS* genes, including *GSC1*, *GSL1,* and *GSL2*, or of *CHS* genes, including *CHS1*, *CHS2*, *CHS3*, *CHS4*, *CHS5*, *CHS6*, *CHS7*, and *CHS8*. Among the four *CHT* genes, *CHT2* and *CHT3* had higher expressions (Fig. 4, top panel). Among the calcineurin-Crz1 signal pathway genes, *CMP1* and *CNB1* were not differentially expressed. Expression of *CRZ1* was not detected. Among the PKC pathway genes, *RLM1* was downregulated. The other genes were not differentially expressed, including *PKC1*, *BCK1*, *MKK2*, *MKC1*, *SWI4*, and *SWI6* (Fig. 4, middle panel). Among the heat shock protein genes, *HSP12*, *HSP70*, *HSP78*, and *HSP104* had higher expressions at 30°C than at 37°C, while the other heat shock protein genes were not differentially expressed, including *HSP21*, *HSP30*, *HSP31*, *HSP60*, and *HSP90* (Fig. 4, bottom panel).

We also compared proteomes of log phase cells of SC5314 incubated at 30°C and at 37°C. Unexpectedly, cells did not exhibit significantly different abundance of proteins encoded by the abovementioned *FKS*, *CHS*, *CHT*, calcineurin-Crz1 signal pathway, or PKC pathway genes. Among the Hsp proteins, Hsp106, Hsp60, Hsp90, Hsp21, and Hsp78 were significantly more abundant in cells grown at 37°C than at 30°C (Table S3).

Taken together, lower CSP susceptibility at 30°C than at 37°C is not due to elevated expressions of *FKS* or *CHS* genes or to decreased expression of *CHT* genes. It is also not due to activation of PKC and calcineurin-Crz1 signaling pathways or higher expression of *HSP90*. It is probably due to altered expression and abundance of other heat shock protein genes such as *HSP12*, *HSP70*, *HSP78*, and *HSP104*.

## DISCUSSION

Echinocandins are the newest antifungal drugs licensed for clinical use. Molecular mechanism of development of resistance to echinocandins has been extensively studied. However, little is known about the impact physiological factors on antifungal efficacy of echinocandins. The normal human body temperature ranges from 36.5°C to 37.5°C. Studies evaluating antifungal activities of echinocandins in *Candida* spp typically use either 30°C (38) or 37°C (39, 40). To the best of our knowledge, our study is the first to investigate the effect of different temperature on the extent of CSP resistance in *C. albicans*.

Consistent with previous studies, deletions of PKC pathway genes *MKK2* and *MKC1* and calcineurin-*Crz1* signaling pathway genes *CMP1* and *CNB1* caused hypersensitivity to CSP (29, 30, 41, 42). Furthermore, we demonstrated that PKC pathway was not required for temperature-modulated CSP resistance. Like the wild type, both *mkk2Δ/Δ* and *mkc1Δ/Δ* strains were still more resistant to CSP at 30°C than at 37°C. We further demonstrated that both pharmacological inhibition and genetic deletion of calcineurin subunits abolished the temperature-modulated CSP resistance; however, strains with deletion of CRZ1 were still more resistant to CSP at 30°C than at 37°C, indicating that CSP resistance modulated by temperature is dependent on calcineurin but is independent on Crz1.

Unexpectedly, cells grown at 30°C and 37°C do not have altered expression of some canonical genes associated with CSP resistance, such as *FKS*, *CHS*, or *CHT* genes, or the PKC and calcineurin pathway genes, or *HSP90*. Several other genes encoding heat shock proteins were highly expressed (1.7–6.4 fold), including *HSP12*, *HSP70*, *HSP78*, and *HSP104*. Heat shock proteins exist in most organisms and have multiple broad functions. In *C. albicans*, there are nine kinds of heat shock proteins with varying molecular sizes: *HSP12*, *HSP21*, *HSP30*, *HSP31*, *HSP60*, *HSP70*, *HSP78*, *HSP90*, and *HSP104*. Calcineurin is a client protein of Hsp90. Requirement of calcineurin in resistance to azoles and echinocandins is dependent on Hsp90 (30, 43). Hsp70 is involved in the transfer of client proteins to Hsp90 for their subsequent activation via the Hsp90 chaperone cycle (44). Hsp12 is a small heat shock protein. Hsp12 usually binds denatured proteins with high affinity until Hsp70 reactivates them (45). In *C. albicans*, overexpression of *HSP12* causes hypersensitivity to azoles (46). In *Cryptococcus neoformans*, deletion of *HSP12* confers hypersensitivity to amphotericin B but not to azoles (47). In *Saccharomyces cerevisiae*, *HSP12* is involved in response to multiple stresses including antifungal drugs (48), and deletion of *HSP12* results in reduced plasticity and flexibility of cell wall (49). *HSP12* and *HSP70* are promising potential targets for antifungal drug development (50). The function of Hsp104 is similar to Hsp70 (51). Hsp78 is a mitochondrial heat shock protein. Hsp78 is implicated in the proteolysis required for the efficient degradation of substrate proteins in mitochondria (52). We posit differential expressions of Hsp12 and Hsp70 at 30°C and 37°C and potentiated calcineurin-dependent CSP resistance.

In conclusion, this study demonstrated a temperature-modulated, non-canonical mechanism of CSP susceptibility in *C. albicans*. Coordinated actions of Hsp12 and Hsp70 probably underlay the essentiality of calcineurin in this novel mechanism.

## Data Availability

Sequence data are available in the ArrayExpress database at EMBL-EBI (www.ebi.ac.uk/arrayexpress) under accession number E-MTAB-11924.

## SUPPLEMENTAL MATERIAL

Fig. S1 spectrum.01790-23-s0001.tif Growth curves of strains with deletions of the calcineurin-Crz1 pathway genes.

Fig. S2 spectrum.01790-23-s0002.tif Control experiment of cyclosporin A dosage effect.

Supplemental legends spectrum.01790-23-s0003.docx Legends for supplemental figures and tables.

Table S1 spectrum.01790-23-s0004.docx Strains used in this study.

Table S2 spectrum.01790-23-s0005.docx Expression of genes induced by CSP at 30°C and 37°C.

Table S3 spectrum.01790-23-s0006.csv Differential proteins at 30°C and 37°C.

ASM does not own the copyrights to Supplemental Material that may be linked to, or accessed through, an article. The authors have granted ASM a non-exclusive, world-wide license to publish the Supplemental Material files. Please contact the corresponding author directly for reuse.
