# Supplementary figures and images for: Susceptibility to caspofungin is regulated by temperature and is dependent on calcineurin in Candida albicans
Source: Microbiol Spectr. 2023 Nov 15;11(6):e01790-23. doi: 10.1128/spectrum.01790-23 (PMC10715083; doi:10.1128/spectrum.01790-23)

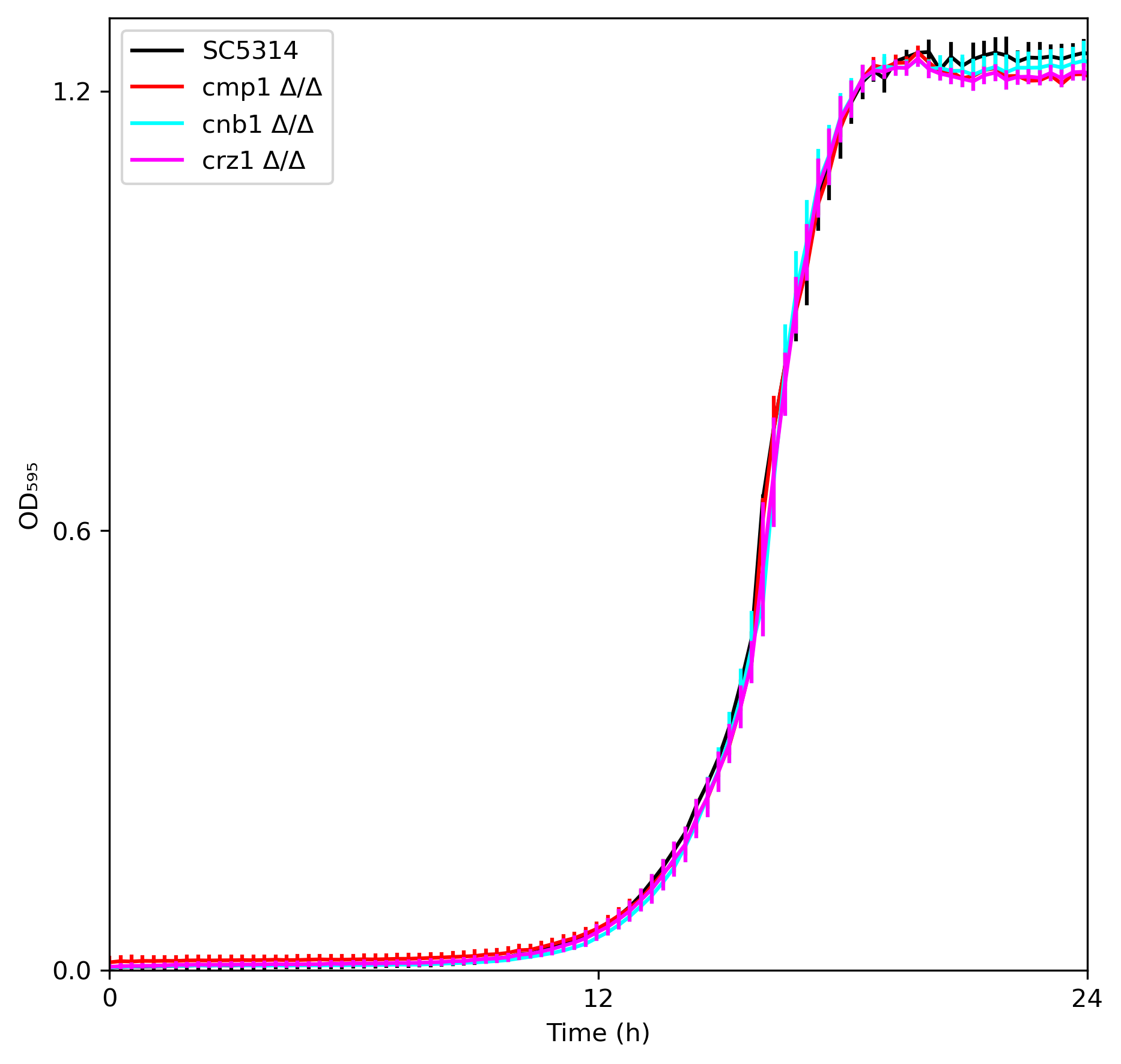

Supplement: Fig. S1 — Growth curves of strains with deletions of the calcineurin-Crz1 pathway genes. [file spectrum.01790-23-s0001.tif]

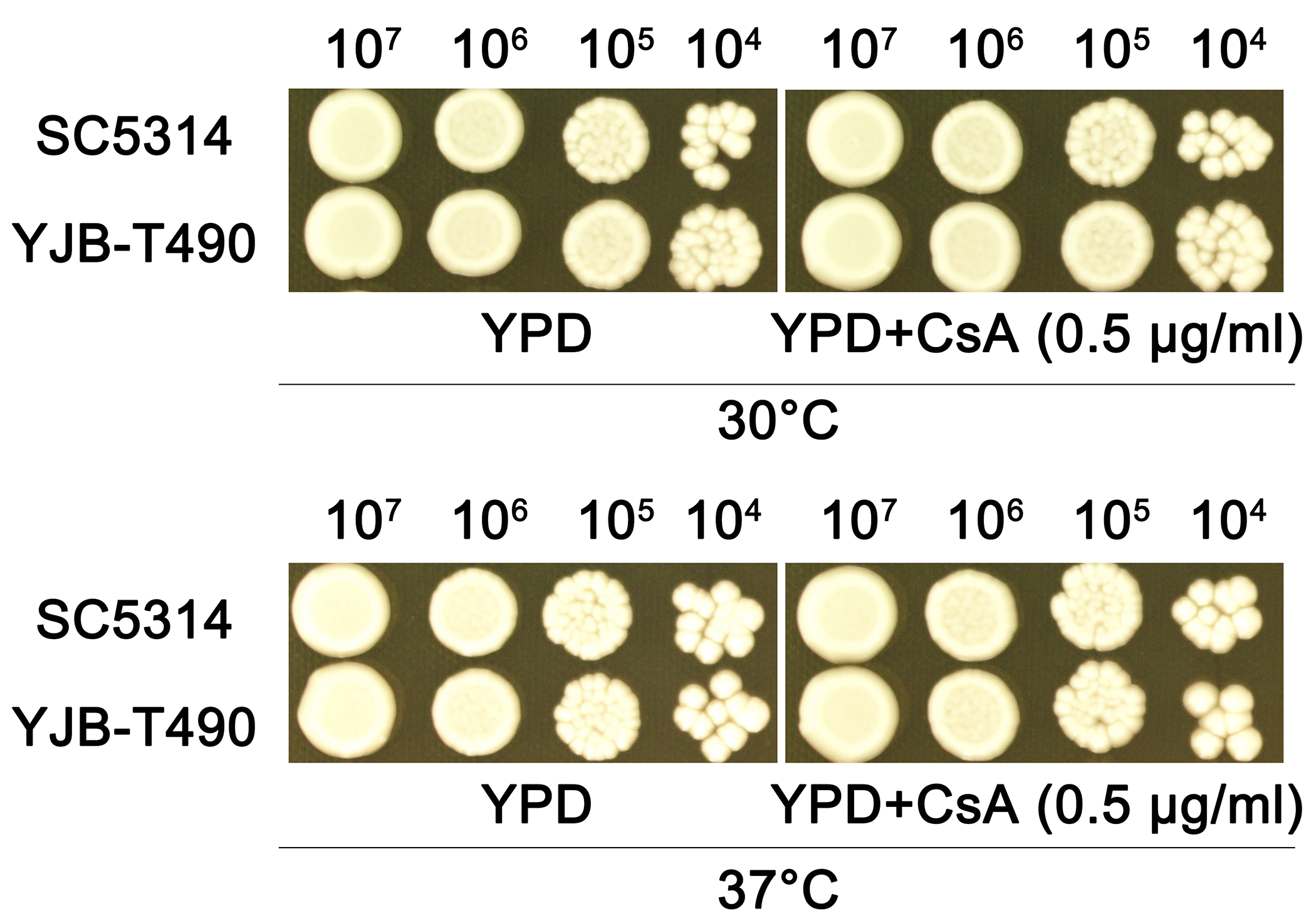

Supplement: Fig. S2 — Control experiment of cyclosporin A dosage effect. [file spectrum.01790-23-s0002.tif]
