# Supplementary material for: Susceptibility to caspofungin is regulated by temperature and is dependent on calcineurin in Candida albicans
Source: Microbiol Spectr. 2023 Nov 15;11(6):e01790-23. doi: 10.1128/spectrum.01790-23 (PMC10715083; doi:10.1128/spectrum.01790-23)
Supplement: Supplemental legends — Legends for supplemental figures and tables. [file spectrum.01790-23-s0003.docx]

**Supplemental materials**

**Figure S1. Growth curves of strains with deletions of the calcineurin-Crz1 pathway genes**

Strains with homozygous deletions of *CMP1*, *CNB1* and *CRZ1* were compared to wild type strain SC5314. The growth was measured in a 96-well plate using a Tecan plate reader. Growth was monitored every 15 min at 30°C for 24 h. Data are represented as the mean ± SD of three biological replicates.

**Figure S2. Control experiment of cyclosporin A dosage effect**

Two wild type strains, SC5314 and YJB-T490 were tested with spot assay for the ability to grow in the presence of 0.5 μg/ml cyclosporin A. The plates were incubated at 30°C (left panel) and 37°C (right panel) for 48h then photographed.

**Table S1. Strains used in this study.**

**Table S2. Expression of genes induced by CSP at 30°C and 37°C**

**Table S3. Differential proteins at 30°C and 37°C**
