# Supplementary material for: Susceptibility to caspofungin is regulated by temperature and is dependent on calcineurin in Candida albicans
Source: Microbiol Spectr. 2023 Nov 15;11(6):e01790-23. doi: 10.1128/spectrum.01790-23 (PMC10715083; doi:10.1128/spectrum.01790-23)
Supplement: Table S1 — Strains used in this study. [file spectrum.01790-23-s0004.docx]

Table S1. Strains used in this study

| Strain | Genotype | Parent | Source |
| --- | --- | --- | --- |
| SC5314 | Wild type |  | (1) |
| YCA892 | mkk2::FRT/mkk2::NAT1 flp | SC5314 | (1) |
| YCA1127 | mkc1::FRT/mkc1::NAT1 flp | SC5314 | (1) |
| YCA641 | cmp1::FRT/cmp1::NAT1 flp | SC5314 | (2) |
| YCA623 | cnb1::FRT/cnb::NAT1 flp | SC5314 | (2) |
| YCA736 | crz1::FRT/crz1::NAT1 flp | SC5314 | (2) |
| YJB-T490 | Wild type | YJB-T490 | (3) |
| YCA748 | cmp1::FRT/cmp1::NAT1 flp | YJB-T490 | This study |
| YCA747 | cnb1::FRT/cnb::NAT1 flp | YJB-T490 | This study |
| YCA749 | crz1::FRT/crz1::NAT1 flp | YJB-T490 | This study |

**Reference**

1. Yang, F., V. Gritsenko, Y. Slor Futterman, L. Gao, C. Zhen, H. Lu, Y.Y. Jiang, and J. Berman, Tunicamycin Potentiates Antifungal Drug Tolerance via Aneuploidy in Candida albicans. mBio, 2021. 12(4): p. e0227221.

2. Xu, Y., H. Lu, S. Zhu, W.Q. Li, Y.Y. Jiang, J. Berman, and F. Yang, Multifactorial Mechanisms of Tolerance to Ketoconazole in Candida albicans. Microbiol Spectr, 2021. 9(1): p. e0032121.

3. Yang, F., E.F.C. Scopel, H. Li, L.L. Sun, N. Kawar, Y.B. Cao, Y.Y. Jiang, and J. Berman, Antifungal Tolerance and Resistance Emerge at Distinct Drug Concentrations and Rely upon Different Aneuploid Chromosomes. mBio, 2023: p. e00227-23.
