# Supplementary material for: Susceptibility to caspofungin is regulated by temperature and is dependent on calcineurin in Candida albicans
Source: Microbiol Spectr. 2023 Nov 15;11(6):e01790-23. doi: 10.1128/spectrum.01790-23 (PMC10715083; doi:10.1128/spectrum.01790-23)
Supplement: Table S2 — Expression of genes induced by CSP at 30°C and 37°C. [file spectrum.01790-23-s0005.docx]

Table S1. Caspofungin regulated genes at 30°C and 37°C

| Gene | 30°C | | 37°C | |
| --- | --- | --- | --- | --- |
|  | Allele A | Allele B | Allele A | Allele B |
| GSC1 | 2.75 | 2.47 | 2.94 | 2.83 |
| GSL1 | 0.93 | 0.74 | 1.13 | 0.86 |
| GSL2 | 0.84 | 0.84 | 1.20 | 1.20 |
| CHS1 | 0.84 | 0.82 | 0.90 | 0.83 |
| CHS2 | 7.18 | 7.18 | 6.10 | 6.10 |
| CHS3 | 3.10 | 2.91 | 2.27 | 1.96 |
| CHS4 | 1.29 | NA | 2.77 | NA |
| CHS5 | 1.25 | 1.03 | 1.53 | 1.16 |
| CHS6 | 0.95 | 0.88 | 1.41 | 0.93 |
| CHS7 | 2.44 | 2.11 | 1.91 | 2.16 |
| CHS8 | 1.61 | 1.46 | 1.86 | 2.22 |
| CHT1 | 1.95 | 1.95 | 1.32 | 1.32 |
| CHT2 | 2.11 | 0.89 | 1.94 | 1.00 |
| CHT3 | 0.12 | 0.12 | 0.19 | 0.19 |
| CHT4 | 1.64 | 2.04 | 2.41 | 1.55 |
| CMP1 | 1.54 | 1.54 | 1.77 | 1.84 |
| CNB1 | 0.86 | 1.07 | 0.83 | 0.77 |
| CRZ1 | 5.12 | 5.12 | 4.22 | 4.22 |
| PKC1 | 0.79 | 0.79 | 0.92 | 0.92 |
| BCK1 | 1.68 | 1.34 | 1.01 | 1.16 |
| MKK2 | 0.98 | 1.11 | 1.16 | 1.26 |
| MKC1 | 1.24 | 1.24 | 0.86 | 0.86 |
| SWI4 | 0.94 | 0.87 | 1.02 | 1.00 |
| SWI6 | 1.22 | 1.27 | 0.99 | 1.63 |
| RLM1 | 1.59 | 1.65 | 1.41 | 1.23 |
| HSP90 | 0.97 | 0.97 | 1.07 | 1.07 |
| HSP78 | 0.82 | 0.82 | 1.38 | 1.23 |
| HSP21 | 0.16 | 0.24 | 0.48 | 0.39 |
| HSP30 | 0.86 | 1.11 | NA | NA |
| HSP31 | 0.68 | 0.25 | 1.03 | 0.98 |
| HSP70 | 0.18 | 0.17 | 0.73 | 0.73 |
| HSP60 | 1.18 | 1.24 | 1.19 | 1.18 |
| HSP104 | 0.28 | 0.25 | 0.89 | 0.75 |
| HSP12 | 0.11 | 0.11 | 0.33 | 0.37 |
